# Strategy to Predict High and Low Frequency Behaviors Using Triaxial Accelerometers in Grazing of Beef Cattle

**DOI:** 10.3390/ani11123438

**Published:** 2021-12-02

**Authors:** Rafael N. Watanabe, Priscila A. Bernardes, Eliéder P. Romanzini, Larissa G. Braga, Thaís R. Brito, Ronyatta W. Teobaldo, Ricardo A. Reis, Danísio P. Munari

**Affiliations:** 1 Departamento de Engenharia e Ciências Exatas, Universidade Estadual Paulista, Jaboticabal 14884-900, São Paulo, Brazil; rafael.nakamura@unesp.br (R.N.W.); larissa.graciano@unesp.br (L.G.B.); 2 Departamento de Zootecnia e Desenvolvimento Rural, Universidade Federal de Santa Catarina, Florianópolis 88034-000, Santa Catarina, Brazil; priscila.arrigucci@ufsc.br; 3 Departamento de Zootecnia, Universidade Estadual Paulista, Jaboticabal 14884-900, São Paulo, Brazil; elieder.romanzini@gmail.com (E.P.R.); zootec.thaisribeiro@hotmail.com (T.R.B.); ronyattaweich@hotmail.com (R.W.T.); ricardo.reis@unesp.br (R.A.R.)

**Keywords:** machine learning, Naïve Bayes Classifier, Nelore, Random Forest, Support Vector Machine

## Abstract

**Simple Summary:**

Monitoring animal activity in production systems is an important tool for obtaining information on health, production, and reproduction. In this study, we evaluated the use of accelerometers with different strategies to predict the grazing behavior of Nelore cattle. This research was conducted in an environment both more challenging and representative of the practices adopted in livestock production systems in Brazil. The results of this study showed that the use of the Random Forest algorithm, together with techniques for resampling the training data of the models, classified the studied behaviors with high accuracy, especially for important, and less frequent activities such as water consumption frequency.

**Abstract:**

Knowledge of animal behavior can be indicative of the well-being, health, productivity, and reproduction of animals. The use of accelerometers to classify and predict animal behavior can be a tool for continuous animal monitoring. Therefore, the aim of this study was to provide strategies for predicting more and less frequent beef cattle grazing behaviors. The behavior activities observed were grazing, ruminating, idle, water consumption frequency (WCF), feeding (supplementation) and walking. Three Machine Learning algorithms: Random Forest (RF), Support Vector Machine (SVM) and Naïve Bayes Classifier (NBC) and two resample methods: under and over-sampling, were tested. Overall accuracy was higher for RF models trained with the over-sampled dataset. The greatest sensitivity (0.808) for the less frequent behavior (WCF) was observed in the RF algorithm trained with the under-sampled data. The SVM models only performed efficiently when classifying the most frequent behavior (idle). The greatest predictor in the NBC algorithm was for ruminating behavior, with the over-sampled training dataset. The results showed that the behaviors of the studied animals were classified with high accuracy and specificity when the RF algorithm trained with the resampling methods was used. Resampling training datasets is a strategy to be considered, especially when less frequent behaviors are of interest.

## 1. Introduction

Monitoring and accessing animal behavior are important tasks in ensuring the success of an animal production system. The animal’s behavior monitored individually and continuously can serve as an indication of its welfare and health [1]. Rumination and feeding behaviors of dairy cattle can indicate productivity measures [2]. How much time animals spend lying down can help estrus detection in cows [3]. By observing how animals walk or how much time they spend lying down can also help to detect and prevent lameness [4]. However, monitoring animal behavior is often carried out by human observation or video monitoring, which makes it difficult to obtain data, due to the demand for human resources [5], as well as the fact that sometimes access to the animals is not easy [6]. Therefore, using accelerometers that automatically measure the animal’s activity has the potential to obtain this information, especially in extensive systems, where access to the animals is more difficult.

There are few studies on systems that help to classify less frequent behaviors such as drinking [7], although these behaviors are indicative of animal health and growth [8]. Drinking behavior has been reported in studies with a controlled environment [9] and in general the authors suggested further studies and adaptations for better drinking behavior predictions [10]. Additionally, the majority of results reported for animal behavior prediction using accelerometer information is with a taurine genetic composition [11,12,13,14], which can be different from a zebu genetic composition, due to the differences in temperament [15].

Less frequent behavior classes can lead to a bottleneck in the classification algorithms’ performance [16]. These less frequent types of behavior increase detection difficulty due to the infrequency and casualness, which results in misclassification of these classes [17]. They can lead to imbalanced data, which refers to a dataset with one or some of the classes having a greater number of observations than the others. The most prevalent class is called the majority class, while the class with less frequent observation is called the minority class [18]. To deal with imbalanced datasets, some resampling techniques are used to rebalance the number of observations in order to facilitate the effect of skewed class distribution in the learning process of a prediction algorithm. These resampling methods are more versatile as they are independent of the classification algorithm [19].

Therefore, the aim of this study was to provide strategies for predicting more and less frequent bovine behaviors, using over and under-sampling training data and comparing three distinct Machine Learning classification algorithms, using information from triaxial accelerometers on pasture raised animals.

## 2. Materials and Methods

All the procedures used followed the Ethical Principles for Animal Experimentation stated by the National Council for Animal Experiment Control and were approved by the Ethics Committee for Use of Animals (CEUA) of Universidade Estadual Paulista (Unesp), under protocol #001081/2019.

### 2.1. Experimental Area and Animals

The experiment was carried out in the Forage Crops and Grasslands section of Universidade Estadual Paulista (Unesp), Jaboticabal, São Paulo, Brazil. The total area was divided into 24 paddocks, seeded with *Trochlea brizantha* (Hochs tex A. Rich) Stapf cv. Marandu (Marandu grass) in 2001. The grazing mean height was 25 cm, using the continuous grazing method with a variable stocking rate. The region’s climate was humid subtropical, with dry winters and rainy summers. The average annual temperature was 22.3 °C, with a maximum average of 29.1 °C and a minimum average of 16.9 °C. The annual average relative humidity was 71.2% and the wettest quarter of the year was the first one (January, February, and March), with an average rainfall of 628.8 mm, equivalent to 44.2% of the total annual rainfall. In this experiment, eight Nelore (*Bos indicus*) animals (343 ± 27 kg), were finished in pastures and were provided a high level of supplementation. The quantity of dietary supplementation provided daily, to reach the animals’ requirements corresponded to 2% of the animals’ body weight, During the dry season. ambient average temperature, maximum average temperature, and minimum average temperature were 26.1 °C, 34.0 °C and 18.5 °C, respectively, and the rainfall was 156.8 mm, distributed over 12 days.

The animals were previously adapted to using tags coupled to custom halters. The tags attached to the halters were kept on the animals (Supplementary Figure S1) for 28 days, which corresponded to the periods of adaptation (25 days) and behavioral observation of the animals. The accelerometers were adjusted on the halter so that it was possible to obtain the information accurately but without causing physical harm to the animals. The animals were observed daily during supplementation and, after the end of the experiment, were evaluated in the corral.

### 2.2. Accelerometers and Animal Behavior

The tags used in this experiment were provided by the Ovi-Bovi^®^ company (Minsk, Belarus) and consist of triaxial accelerometers using a microelectromechanical system (MEMS) (model LIS2DE12; ST Microelectronics^®^ (Plan-les-Ouates, Switzerland)), weighing 80 g, with dimensions of 105 mm × 60 mm × 22 mm, and attached to a custom halter and placed on the underjaw of young bulls to detect their movements (Figure S1). The accelerometers provide movement information along three axes: (X [horizontal movements—side to side], Y [longitudinal movements—front to back] and Z [vertical movements—up and down]). The information from the accelerometers was transmitted in 6 s window size (approximately 0.167 Hz) time intervals and collected through a wireless system (band of 433 MHz) and later stored in the cloud (Ovi-Bovi^®^ company (Minsk, Belarus) server). The window size was determined following the manufacturer’s recommendations and based on previous results, taking into account the biology of the behaviors studied, battery life and data loss due to their collision at the time of transmission.

The behavioral observations of the animals were carried out during a 12 h per day period (6 a.m. to 6 p.m.) for two consecutive days (24 September 2019 and 1 October 2019). The animal’s behavior was noted whenever the animal changed its behavior, registering the time when it occurred. The behavior activities observed were grazing, ruminating (noted whenever rumination was observed, whether standing or lying down), idle (lying or standing), water consumption frequency (WCF), feeding (supplementation) and walking. A description of each behavior is provided in Supplementary Table S1. When the animal changed behavior, the time was noted according to each activity by the animal.

### 2.3. Data Processing and Prediction Algorithms

All accelerometer data was processed using the R base package (version 4.0.0, RStudio, Boston, MA, USA) [20]. The raw data from the accelerometers was accessed by the Ovi-Bovi^®^ (Minsk, Belarus) tag provider server. This data consisted of tag identification, information of time and date and the variables of the movement axes, x, y and z (transformed into gravity unit *g* = 9.18 m s^−2^), which totalized 101,144 records for each variable. In addition to the variables provided by the accelerometers, the predictor variables of signal magnitude area (SMA), signal vector magnitude (SVM), movement variation, energy, entropy, pitch, roll, and inclination were calculated based on information from the three movement axes, according to Alvarenga et al. [21]. The equations for the calculations of these variables are presented in Supplementary Table S2. Additionally, the meteorological variables provided by the agroclimatological station of Universidade Estadual Paulista (Unesp), Jaboticabal, São Paulo, Brazil were considered as predictors in the models. The weather station was located 800 m away from the experimental site. The variables were air temperature, relative humidity, wind speed, wind direction, solar radiation, and maximum wind gust, provided in 10 min daily time intervals.

The prediction of animal behaviors through accelerometer information was evaluated using Random Forest (RF) [22], Support Vector Machine (SVM) [23] and Naïve Bayes Classifier (NBC) [24] algorithms. The first algorithm, RF, was performed by the randomForest R package [25] considering 500 trees (ntree), five variables randomly sampled as candidates at each split (mtry) with the predictors’ importance being taken into account (importance). The second and third algorithms were performed by the e1071 R package [26]. The SVM models were performed using a classification method type, a radial kernel type with a 0.1 g value and a cost of constraints violation of 10. The NBC models were built with default function arguments. All the algorithms considered the raw accelerometer data, transformed into gravity units, calculated variables as mentioned and meteorological variables as predictors and the animals’ behaviors, as a response variable. Window size considered for predictions was 6 s with no overlapping window stride.

The dataset was divided into training (70% of the original dataset), wherein the predictor variables and all the observed animal behavior were considered, and test (30% of the original dataset) datasets, wherein only the predictor variables were included. The accelerometer data were compared on a 6 s basis with the observation data. Each algorithm was trained to classify the six behaviors considered. As the observations data were carried out by noting the time that the animal changed its behavior and the window size of each event recorded by the accelerometer was 6 s, the observation data was replicated until the behavior change to compare with the accelerometer data. To compare the prediction ability of each model, the sensitivity (1), specificity (2), accuracy (3), and Kappa coefficient [27], which compares the observed accuracy with the expected accuracy (random chance), were calculated for the test dataset using the confusionMatrix function of caret R package [28].

(1) $$\mathrm{sensitivity}=\frac{\mathrm{true}\;\mathrm{positive}}{\left(\mathrm{true}\;\mathrm{positive}+\mathrm{false}\;\mathrm{negative}\right)}$$

(2) $$\mathrm{specificity}=\frac{\mathrm{true}\;\mathrm{negative}}{\left(\mathrm{true}\;\mathrm{negative}+\mathrm{false}\;\mathrm{positive}\right)}$$

(3) $$\mathrm{accuracy}=\frac{\left(\mathrm{true}\;\mathrm{positive}+\mathrm{true}\;\mathrm{negative}\right)}{\left(\mathrm{true}\;\mathrm{positive}+\mathrm{true}\;\mathrm{negative}+\mathrm{false}\;\mathrm{positive}+\mathrm{false}\;\mathrm{negative}\right)}$$

where true positive was the number of instances in which the animal behavior of interest was correctly classified after testing; false negative was the number of instances in which the animal behavior of interest was observed visually but was classified incorrectly as some other animal behavior; false positive was the number of instances in which the animal behavior of interest was incorrectly classified but not observed; and true negative was the number of instances in which the animal behavior of interest was correctly classified as not being observed.

### 2.4. Resampling Methods to Deal with Imbalanced Data

**Figure 1:**
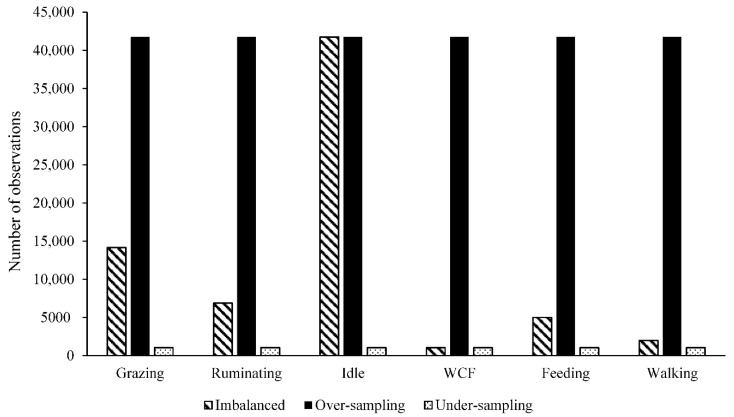
Number of observations for the imbalanced, over and under-sampled training datasets for grazing, ruminating, idle, WCF, feeding and walking behaviors in grazing beef cattle.

To deal with an imbalanced dataset (Figure 1) that can impair the predictive ability of the studied methods, two resampling methods were used in the training dataset. The over-sampling method [29], which eliminated the damage caused by skewed distribution by creating new minority class samples, and the under-sampling method [30], which also eliminated the damage caused by skewed distribution, but by removing the intrinsic samples in the majority class. The functions upSample and downSample from the caret R package [28] were used to add additional samples to the minority classes with replacements to make the class distributions equal and to discard samples randomly so that all classes had the same frequency as the minority class, respectively.

## 3. Results

In general, overall accuracy was higher for RF models, being the greatest for the RF model trained with over-sampled data. The lowest overall accuracy was observed in the NBC model trained with over-sampled records, which was the only method in which the over-sampling showed negative effects on behavior classification, since for the RF and NBC algorithms the training with over-sampled data promoted the highest results (Table 1). The same patterns can be observed for the Kappa coefficient, where the highest values were for the RF algorithm and, with the exception of the NBC algorithm, the highest values were observed when the training was performed with over-sampled data.

The proportions for each behavior of the total observations were equal to 20%, 10%, 59%, 1%, 7% and 3% for grazing, ruminating, idle, WCF, feeding and walking, respectively. The greatest sensitivity (0.808) for the less frequent behavior (WCF) was observed in the RF algorithm trained with under-sample data (0.808), followed by the same algorithm trained with over-sample and imbalanced data (0.590). Similar results were noted for the second less frequent behavior (walking). Feeding behavior presented a greater proportion of true positive observations when the RF with over-sampled training was used (0.860), followed by under-sampled (0.768) and imbalanced (0.688) training in the same method.

Considering the NBC algorithm to classify feeding behavior, training with re-sampled records showed better results than training with imbalanced data, with a difference ranging from 0.255 to 0.297 for sensitivity (Table 1). Classifying ruminating behavior by the RF algorithm was better for re-sampled training, however, the training with imbalanced data also presented a high sensitivity. The NBC algorithm showed higher sensitivity prediction for ruminating behavior when training datasets were with over-sampled (0.865) and imbalanced (0.700) records, followed by the under-sample records (0.580). Grazing and Idle behaviors resulted in a greater proportion of true positive observations in the trained over-sampled and imbalanced RF algorithm, followed by the RF algorithm trained with under-sample data (Table 1).

The SVM models only performed efficiently when classifying the two more frequent behaviors (imbalanced and over-sampled training datasets for idle and under-sampled training dataset for grazing). The lowest proportion of true negative observations was found in SVM models when classifying the most frequent behavior (idle) with imbalanced and over-sampled training datasets (0.059 for both models), followed by the classification for grazing behavior using the under-sampled training dataset (0.120).

## 4. Discussion

The highest overall accuracy values observed for the RF algorithm (Table 1) corroborate the accuracy results, comparing RF with other Machine Learning algorithms, found in a study that classified behaviors in wild animals [31] and human behavior [32] using accelerometer information. The RF classification algorithm is highly capable at selecting and classifying predictor variables and at discriminating between predicted variables. This RF feature becomes important to evaluate information derived from accelerometers because these generate large amounts of data, which consume more time to select relevant variables [33] and lead to error prone and subjective tasks [34]. Therefore, due to the greater stability compared to SVM [35], the overall accuracy results of the current study were higher when RF was used.

When compared to SVM, the NBC algorithm showed the lowest overall accuracies. This lower result was also found in a study classifying cows’ behavior, comparing classification algorithms, using accelerometer information [36]. The overall accuracy result for the SVM algorithm of the present study (ranging from 0.267 to 0.611) was lower than that observed by the aforementioned study. According to Douglas et al. [37], SVM algorithms can be more suitable for complex classification tasks, especially in the training algorithm process. When trying to classify sow-activity using accelerometer data, Escalante et al. [38] found the lowest performance using the NBC algorithm, compared to SVM and RF. In our study, the SVM algorithm did not perform well, in general, when classifying the behaviors studied, as observed by the aforementioned authors.

Due to the short period of time and intermittence that cattle drink water [39,40], this behavior tends to be less frequent than other observed behaviors and often this action is not considered in the classification analyses, even if this behavior was observed and noted [12,41]. In the current study, the less frequent behavior of WCF presented better results than those found in the literature [10], when the RF algorithm was used, especially when trained with resampled datasets. Similarly, when classifying WCF using resampled training and the RF algorithm, the sensitivity and specificity were greater than those found by Williams et al. [9], assessing the classification of drinking water behavior in cattle in periods of time less than or equal to 10 s. Although these authors reported higher true positive rate results when time periods longer than 10 s were observed, the experiment was conducted in a more controlled environment and used, in addition to accelerometers, a water flow meter. The results found in the present study were obtained in an extensive rearing system environment, a widely used practice, therefore representative of Brazilian regions and of the greater difficulty in handling the animals. The better results for predicting lower frequency behaviors, found in the current study, may help in future studies to monitor animals’ health and welfare and also in genetic breeding programs.

Performance in predicting true positives for feeding and walking behaviors using NBC and SVM algorithms with the three training datasets considered was higher than the percentage of correct classifications in a study classifying sow-activity using accelerometer data [38]. When the RF algorithm was considered, walking activity had better prediction results when resampling methods were used. Feeding behavior had a higher correct classification when all three RF datasets were used. Even in a more controlled environment, with more data collected and the animals’ behavior being monitored by video cameras, the results of the present study were, in general, greater than those found in the aforementioned study. The resampling strategy used in this paper can lead to better behavior classifications.

Resampling training with the RF algorithm showed higher sensitivity results than those observed with unbalanced data in predicting rumination, WCF, feeding and walking activities. The same was observed when the NBC algorithm was considered, as well as for prediction of rumination, WCF and walking with the under-sampled dataset in the SVM algorithm. The slight decrease or no sensitivity gain observed for grazing and idle behaviors when the resample methods were used, especially for RF and NBC, may have occurred due to the random sampling of these majority behavior classes, leading to a decrease in their true positive rates. Balancing the database to equalize the number of observations should only be performed when the class of interest is the minority one [42]. Idle behavior had higher sensitivity results when considering the imbalanced and under-sampled datasets for the RF algorithm. According to Escalante et al. [38], passive activities are more difficult to classify due to noise generated when eventual animal movements happen. Thus, the RF algorithm can deal better with noisy measurements when more observations for idle behavior are considered.

Considering the SVM algorithm with the imbalanced training dataset, grazing behavior had low sensitivity and high specificity, while for idle behavior the sensitivity was high and the specificity low. This pattern was repeated within each behavior for training with the under and over-sampled datasets. A possible explanation is due to the fact that the SVM algorithm confused the classification of grazing behavior with idle behavior. Martiskainen et al. [43] observed that the SVM algorithm confused some of the behaviors studied, especially in similar activities, according to the position of the accelerometer attached to the cows. The differences observed in each movement pattern of a given behavior can interfere in the movement’s classification [44].

According to Zughrat et al. [45], the under-sampling technique can drastically reduce the number of support vectors in an SVM algorithm, leading to less computational demand, resulting in a performance gain if compared with the over-sampling technique. In the present study, the under-sampled dataset used with SVM, showed higher sensitivity results for the majority of classified behaviors, however for idle behavior this was not observed, probably also due to the confusion in the classification of behaviors, where the algorithm may have classified idle behavior as grazing when the under-sampled training dataset was used. Using this resampling technique, data from the majority class was removed, which may also have influenced this result. The opposite may have occurred for the oversampled and imbalanced training datasets. Using resampling methods in training datasets can promote little or no gain in the predictive performance of this algorithm [46].

When the under-sampling technique was considered, due to the fact that the observations of the majority behavioral classes were reduced, the sensitivity of these classes was impaired. In general, the RF algorithm was the one that best managed to classify the studied behaviors, together with the over-sampling training dataset, as it increases the number of observations for the minority behavior classes without impairing the classification of the majority classes. However, it should be taken into account that when increasing the amount of information there is greater computational cost for the analyses and a greater amount of time is needed to accomplish them.

## 5. Conclusions

The results showed that the behaviors of the studied animals were classified with great accuracy and specificity when the RF algorithm trained with the resampling methods was used. Therefore, in general, the best strategy to classify and predict more frequent behaviors was using the RF algorithm, and when less frequent behaviors are the main interest, the most appropriate strategy would be using the over-sampling technique for training the data.

## Figures and Tables

**Table 1:** Sensitivity, specificity, overall accuracy and Kappa coefficient for the Machine Learning algorithms and resampled training datasets for the studied behaviors.

Algorithm and Resample Training Datasets	Behaviors						Overall Accuracy	Kappa Coefficient
	Grazing	Ruminating	Idle	WCF	Feeding	Walking		
Random Forest								
Imbalanced							0.880	0.789
Sensitivity	0.816	0.876	0.957	0.278	0.688	0.501		
Specificity	0.960	0.996	0.823	0.999	0.991	0.998		
Over-sampling							0.920	0.865
Sensitivity	0.894	0.938	0.952	0.590	0.860	0.700		
Specificity	0.966	0.995	0.917	0.999	0.990	0.997		
Under-sampling							0.647	0.505
Sensitivity	0.644	0.901	0.580	0.808	0.768	0.797		
Specificity	0.892	0.921	0.942	0.948	0.925	0.949		
Support Vector Machine								
Imbalanced							0.611	0.078
Sensitivity	0.039	0.131	0.995	0.021	0.100	0.027		
Specificity	0.998	0.999	0.059	0.999	0.999	0.999		
Over-sampling							0.611	0.078
Sensitivity	0.039	0.131	0.995	0.021	0.100	0.027		
Specificity	0.998	0.999	0.059	0.999	0.999	0.999		
Under-sampling							0.267	0.075
Sensitivity	0.970	0.201	0.066	0.222	0.096	0.204		
Specificity	0.120	0.994	0.992	0.994	0.994	0.992		
Naïve Bayes Classifier								
Imbalanced							0.367	0.100
Sensitivity	0.284	0.700	0.392	0.000	0.105	0.083		
Specificity	0.922	0.605	0.608	0.999	0.971	0.971		
Over-sampling							0.179	0.072
Sensitivity	0.122	0.865	0.065	0.138	0.360	0.157		
Specificity	0.962	0.422	0.963	0.933	0.850	0.950		
Under-sampling							0.362	0.124
Sensitivity	0.121	0.580	0.423	0.015	0.402	0.126		
Specificity	0.958	0.702	0.727	0.993	0.815	0.954		

## Data Availability

The data that support the findings of this study is available upon reasonable request contacting the corresponding author.

## Supplementary Materials

The following are available online at https://www.mdpi.com/article/10.3390/ani11123438/s1, Figure S1: Male Nelore with an accelerometer attached to the custom halter in the mandibular region. Table S1: Description for each animal behavior studied. Table S2: Predictor variables created from x, y, and z axis acceleration variables.
