# Supplementary material for: Strategy to Predict High and Low Frequency Behaviors Using Triaxial Accelerometers in Grazing of Beef Cattle
Source: Animals (Basel). 2021 Dec 2;11(12):3438. doi: 10.3390/ani11123438 (PMC8698044; doi:10.3390/ani11123438)
Supplement: Supplementary file 1 [file animals-11-03438-s001.zip › animals-1434557-supplementary.pdf]

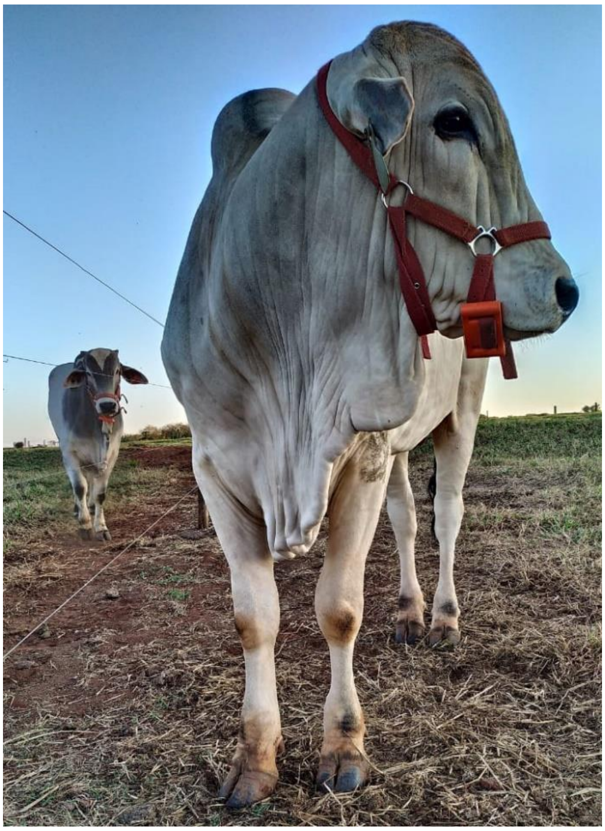

Figure S1. Male Nelore with an accelerometer attached to the custom halter in the mandibular region.

Table S1. Description for each animal behavior studied.

Animal behavior	Description
Grazing	Animals searching for food while walking short distances with their head down, without picking food up with their mouth; standing still with their head down while apprehending food with their mouth; and chewing either with their head down or their head up, while stationary.
Ruminating	Animals chewing and swallowing a ruminal bolus.
Idle	Animals being idle. Lying down in any resting position or standing up on all four legs, without locomotion.
WCF	Animals attending the water fountain.
Feeding	Animals located in the feeding supplement zone, ingesting dietary supplement.
Walking	Animals walking from one position to another, without searching for food or ruminating.

WCF: Water Consumption Frequency.

Table S2. Predictor variables created from x, y, and z axis acceleration variables

Predictor variables	Equation
SMA	$\lvert X_i\rvert + \lvert Y_i\rvert + \lvert Z_i\rvert$
SVM	$\sqrt{X_i^2 + Y_i^2 + Z_i^2}$
Movement variation	$\lvert X_{i+1} - X_i\rvert + \lvert Y_{i+1} - Y_i\rvert + \lvert Z_{i+1} - Z_i\rvert$
Energy	$\left(X_i^2 + Y_i^2 + Z_i^2\right)^2$
Entropy	$\left(1 + (X_i + Y_i + Z_i)\right)^2 x \ln(1 + (X_i + Y_i + Z_i)^2)$
Pitch (degrees)	$\tanh^{-1}\left(-X_i/\left(\sqrt{Y_i^2 + Z_i^2}\right)\right) x\ 180/\pi$
Roll (degrees)	$atan2(Y_i Z_i) x\ 180/\pi$
Inclination (degrees)	$\tan^{-1}\left(\left(X_i^2 + Y_i^2\right)/Z_i\right) x\ 180/\pi$

SMA – signal magnitude area; SVM – signal vector magnitude [22].
